# Pilot Randomized Controlled Trial of a Standard Versus a Modified Low-Phosphorus Diet in Hemodialysis Patients

**DOI:** 10.1016/j.ekir.2020.08.008

**Published:** 2020-08-18

**Authors:** Fiona N. Byrne, Barbara A. Gillman, Mairead Kiely, Brendan Palmer, Frances Shiely, Patricia M. Kearney, Joyce Earlie, Maria B. Bowles, Fiona M. Keohane, Pauline P. Connolly, Sarah Wade, Theresa A. Rennick, Bernice L. Moore, Oonagh N. Smith, Celene M. Sands, Orla Slevin, Denise C. McCarthy, Karina M. Brennan, Halóg Mellett, Darren Dahly, Eoin Bergin, Liam F. Casserly, Peter J. Conlon, Kieran Hannan, John Holian, David W. Lappin, Yvonne M. O'Meara, George J. Mellotte, Donal Reddan, Alan Watson, Joseph Eustace

**Affiliations:** 1 Department of Nutrition and Dietetics, Cork University Hospital, Cork, Ireland; 2 Department of Renal Medicine, Cork University Hospital, Cork, Ireland; 3 Health Research Board, Clinical Research Facility, Cork, Ireland; 4 Department of Nutrition and Dietetics, Mater Misericordiae University Hospital, Dublin, Ireland; 5 Department of Renal Medicine, Mater Misericordiae University Hospital, Dublin, Ireland; 6 School of Food and Nutritional Sciences University College Cork, Cork, Ireland; 7 School of Public Health, University College Cork, Cork, Ireland; 8 Beacon Renal, Tallaght, Dublin, Ireland; 9 Department of Nutrition and Dietetics, University Hospital Limerick, Limerick, Ireland; 10 Departments of Nephrology and Internal Medicine, University Hospital Limerick, Limerick, Ireland; 11 Department of Nutrition and Dietetics, Cavan General Hospital, Cavan, Ireland; 12 Department of Medicine, Cavan General Hospital, Cavan, Ireland; 13 Department of Nutrition and Dietetics, St. Vincent’s University Hospital Dublin, Dublin, Ireland; 14 Department of Nephrology, St. Vincent’s University Hospital Dublin, Dublin, Ireland; 15 Department of Nutrition and Dietetics, Midland Regional Hospital, Tullamore, Ireland; 16 Department of Nephrology, Midland Regional Hospital, Tullamore, Ireland; 17 Department of Nutrition and Dietetics, Beaumont Hospital, Dublin, Ireland; 18 Beaumont Hospital Kidney Center, Beaumont Hospital, Dublin, Ireland; 19 Department of Nutrition and Dietetics, Mayo University Hospital, Mayo, Ireland; 20 Department of Nutrition and Dietetics, Galway University Hospitals, Galway, Ireland; 21 Department of Nephrology, Galway University Hospitals, Galway, Ireland; 22 Northern Cross Dialysis Center, Fresenius Medical Care, Dublin, Ireland; 23 Beacon Renal Drogheda, Drogheda, Ireland; 24 Department of Nutrition and Dietetics, Tallaght University Hospital, Dublin, Ireland; 25 Department of Nephrology, Tallaght University Hospital, Dublin, Ireland

**Keywords:** diet, hyperphosphatemia, phosphates, potassium, renal dialysis

## Abstract

**Introduction:**

The standard low-phosphorus diet restricts pulses, nuts, and whole grains and other high phosphorus foods to control hyperphosphatemia. We conducted a randomized controlled trial to evaluate the effectiveness, safety, and tolerability of the modified diet, which introduced some pulses and nuts, increased the use of whole grains, increased focus on the avoidance of phosphate additives, and introduced the prescription of low-biological-value protein such as bread.

**Methods:**

We conducted a multicenter, pragmatic, parallel-arm, open-label, randomized controlled trial of modified versus standard diet in 74 adults on hemodialysis with hyperphosphatemia over 1 month. Biochemistry was assessed using monthly laboratory tests. Dietary intake was assessed using a 2-day record of weighed intake of food, and tolerability was assessed using a patient questionnaire.

**Results:**

There was no significant difference in the change in serum phosphate between the standard and modified diets. Although total dietary phosphorus intake was similar, phytate-bound phosphorus, found in pulses, nuts, and whole grains, was significantly higher in the modified diet (*P* < 0.001). Dietary fiber intake was also significantly higher (*P* < 0.003), as was the percentage of patients reporting an increase in bowel movements while following the modified diet (*P* = 0.008). There was no significant difference in the change in serum potassium or in reported protein intake between the 2 diets. Both diets were similarly well tolerated.

**Conclusion:**

The modified low phosphorus diet was well tolerated and was associated with similar phosphate and potassium control but with a wider food choice and greater fiber intake than the standard diet.

## Graphical abstract

**Figure:**
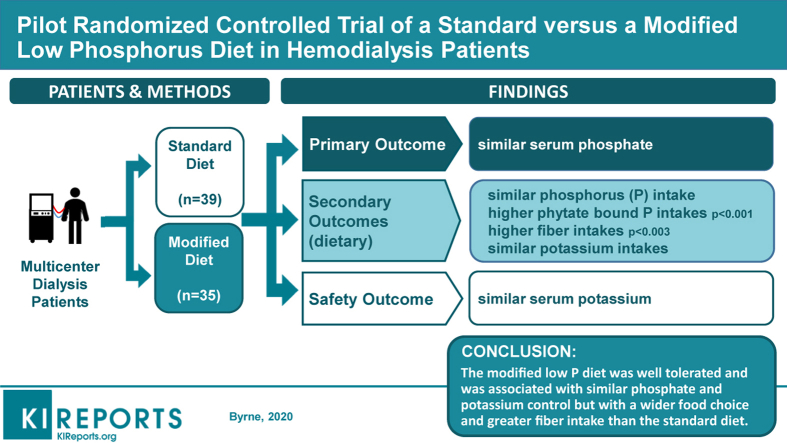

See Commentary on Page 1845

Hyperphosphatemia is common in end-stage kidney disease (ESKD) and is associated with excess morbidity and mortality. Dietary phosphorus restriction is suggested to control hyperphosphatemia.^1^^,^^2^ Traditionally total phosphorus intake is reduced by moderating protein intake, by restricting food with a high phosphorus content (e.g., dairy, whole grains, pulses, and nuts), and by avoiding phosphate additives.

Emerging opinion supports the introduction of more plant protein in the form of whole grains, pulses, and nuts in which the phosphorus is largely bound by phytate and therefore not as available for absorption.^3^, ^4^, ^5^, ^6^, ^7^, ^8^, ^9^, ^10^ Avoidance of additives has been shown to improve phosphate control.^11^ As there is a strong linear relationship between dietary protein and phosphorus intake,^12^ prescription of protein is essential. This is both to ensure that increased protein needs are met, but also to avoid overconsumption of protein that carries an obligatory phosphorus load. It should be possible to ensure adequate protein intake while restricting phosphorus.^13^^,^^14^

Based on this emerging evidence, we revised the Irish national low phosphorus diet sheet. We conducted a multicenter, pragmatic, parallel-arm, open-label, randomized controlled trial (RCT) of the efficacy, safety and tolerability of the modified diet in order to guide implementation.

The primary study objective was to determine whether the modified low phosphorus diet was comparable to current management in reducing serum phosphate levels in hemodialysis (HD) patients. The secondary objectives were to determine self-reported tolerability, safety in respect to hyperkalemia, and the nutritional composition, specifically the phosphorus, the phytate bound phosphorus, and the fiber content of the modified diet.

## Materials and Methods

The protocol for this national, multicenter, pragmatic, parallel-arm, open-label RCT was developed according to the SPIRIT statement^15^ and conducted in accordance with the Declaration of Helsinki. The trial was registered at ClinicalTrials.gov: NCT03146923. Ethical approval was obtained from the Clinical Research Ethics Committee of the Cork Teaching Hospitals ECM 4(I) 10/01/17 and at each center where the trial took place. All participants provided written informed consent.

Practicing registered renal dietitians from all dialysis units in Ireland were invited to participate in the trial. A total of 15 dietitians from 9 university hospital dialysis units and 4 satellite dialysis units agreed to participate. Training, including Good Clinical Practice, general research training and methodology specific to the trial, was provided at a 2-day training program held by the Health Research Board Clinical Research Facility Cork (HRB CRF-C) at University College Cork in January 2017.

Inclusion criteria were: age ≥18 years; a self-reported urine output of <400 ml/d; treatment with hemodialysis for >3 months; and an average serum phosphate of >5mg/dl over the last 3 months. Exclusion criteria were hyperkalemia (predialysis serum potassium of >6 mEq/l in the month preceding the trial); parathyroidectomy; and an abnormal corrected serum calcium level in the preceding month. Patients with acute concurrent illness who required hospitalization in the 2 weeks prior to recruitment were also excluded. Patients with abnormal serum corrected calcium were also excluded, as this may have warranted intervention during the trial.

All participants were receiving standard care, which involved one-to-one counseling with the patient and the patient’s relevant family members or carers/caregivers regarding a diet that provides approximately 1000 mg phosphorus per day and is based on the national diet sheet Eating Well with Kidney Disease.^16^ It includes the following main components: restricting protein intake to requirements of 1 to 1.2 g/kg ideal body weight (IBW); aiming for 70% high biological value (HBV); restricting dairy intake (1−1.5 portions per day equivalent to 200−300 ml of milk); avoiding foods high in phosphate; and avoiding foods with phosphate additives.

The evidence base for the revised dietary phosphorus recommendations and their translation into dietary advice, as well as the diet sheet, have been described in detail elsewhere.^17^^,^^18^ Table 1 summarizes the main changes made to the modified diet. We attempted to blind patients by avoiding the use of terminology such as “old” and “new” and by retyping the standard diet sheet to match the font, layout, and introduction of the modified diet sheet. Both diet sheets were approved by the National Adult Literacy Agency (NALA).

**Table 1:** Summary of changes in modified diet sheet

Nutrient-based recommendations	Food-based recommendations
Inclusion of foods with reduced phosphorus bioavailability due to phytate content	Two of the daily allowances of high biological value protein exchanges (7 g protein/exchange) are replaced with plant-based vegetarian protein exchanges (e.g. replace 50 g of meat with 100 g of pulses and 25 g of unsalted peanuts).Whole grain breads and cereals are encouraged.
Focus on more accurate protein prescription of 1.1 g of protein/kg ideal body weight, thus avoiding overprescription of protein that carries an obligatory protein load, and include some focus on phosphorus-to-protein ratio.	Bread, cereals, and potatoes have been included in prescribed daily protein allowances. Target percentage of protein from high biological value changed from 70% to 50%−70%. Fish has been reduced to 25 g of fish per 7 g of protein exchange. Two portions of fruit and 2 portions of vegetable were counted as 4 g of protein. Phosphorus-to-protein ratio: Beef has lowest P:protein ratio (7 mg/g), with oily fish having the highest ratio (11 mg/g). However, the consensus reached was not to focus on the ratio in this food group, and to broadly follow healthy eating guidelines and to encourage variety, including consumption of fish twice a week. Dairy products are restricted to 1 portion per day P:protein ratio 20−30 mg/g. Egg whites, which have an extremely low ratio 1.1 mg P/g protein, are included
Full avoidance of phosphate additives from the European Union list of authorized phosphate additives in foods.	Check for phosphate E numbers E338, E339,E340, E341, E343, E450, E451, E452, and E541. We also advised to check for “phos” on ingredient lists, giving examples that we commonly encountered on labels, such as diphosphate, sodium polyphosphate, and calcium triphosphate.

P, phosphorus.

From 1 week before the trial began, relevant medications, dialysis times, or dialysate were not changed unless approved by the nephrologists. Prior to randomization, all patients received an individual anthropometric and dietary assessment by a registered renal dietitian. Following randomization and baseline blood tests, patients randomized to standard care were re-educated regarding their diet and provided with the standard diet sheet. Patients following the modified diet were educated and provided with their diet sheet, product samples of pulses and nuts, a recipe booklet, and a shopping card of phosphate additives to look out for. Both groups were given weighing scales to help with portion control, especially for high-protein foods such as meat and fish. Participants were visited on the second and last weeks of the trial to remind them of the importance of adhering to the intervention for the month.

The trial started with education of the participants regarding their assigned diet, following their routine monthly dialysis laboratory tests, which served as baseline blood tests, and ended at the following month’s routine set of laboratory tests, which served as the end of intervention blood tests. In units that had access to a suitable freezer, an additional sample was taken to assess FGF23.

In the days leading up to the end of intervention blood tests, patients kept a 2-day record of weighed intake, including 1 dialysis day and 1 nondialysis day, to assess dietary changes. The days were nonconsecutive days. Participants were educated on how to record the weighed intake and were asked to collect labels so that the researcher could more accurately capture foods that had phosphate additives. If the weight of the food was not available, we used the hierarchical approach to quantification previously described.^19^ Nutrient analysis was carried out using Nutritics Software.^20^ A bespoke additional data field was added to the software to allow the dietitian to tag foods with a significant phytate content, including pulses, nuts and wholemeal/wholegrain breads, cereals, pasta, and rice. Foods identified as having a phosphate additive were also marked on data entry using a second added field. Nutritics contains multiple nutrient databases; however, we restricted the databases used to McCance and Widdowson’s composition of foods integrated dataset,^21^ the USDA Food Composition Databases,^22^ and the Irish food composition databases.^23^ To standardize food coding practice and data entry, the coordinating center provided units with a list of standard food codes for foods generated from the diet sheets, and for any new recipes or new food codes required. Food code queries were answered by the coordinating center, collated, and shared with all centers. Further details are described in the standard operating procedure available online as an Open Science Framework (OSF) project (https://doi.org/10.17605/OSF.IO/FSK8T).

Participants were also asked to complete a tolerance questionnaire. When the trial concluded, each patient was advised to go back to following the standard diet.

There are no core outcome sets for ESKD. Based on prior trials^11^ and audit of phosphate data, it was calculated that we would require a total of 56 participants in each arm, using an independent-sample *t* test with a 1:1 allocation ratio, a 2-sided type I error of 0.05, a power of 90%, and an effect size of 0.73. Power remained at 80% even with 64 analyzable participants (32 in each arm), all else being equal.

The specific randomization list was designed by an independent trial statistician, and participants were randomly assigned to either the control or intervention group with a 1:1 allocation as per a computer-generated randomization schedule, stratified by site and by baseline phosphate, using permuted blocks of random sizes and allocated using a sequentially numbered, opaque, sealed envelope approach. The block sizes were not disclosed so as to ensure concealment. The allocation of patients to treatment arms was revealed after the patient was consented. Patients were withdrawn from the study if they had a confirmed serum potassium of ≥6.3 mEq/l. Statistical analysis was performed by staff at the HRB CRF-C. Categorical data were described as counts and percentages, and continuous variables were summarized using medians and minimum and maximum values. The study outcomes were explored using simple or multiple linear regression. We report estimates, 95% confidence intervals, and corresponding *P* values. Analysis of categorical patient survey data was performed using a Fisher exact test. Analyses were done on an intention-to-treat basis. All analyses were conducted using the R Project for Statistical Computing version 3.6 (R Core Team, Vienna, Austria; www.R-project.org/). The analysis code is available online as an OSF project (https://doi.org/10.17605/OSF.IO/FSK8T).

## Results

**Figure 1:**
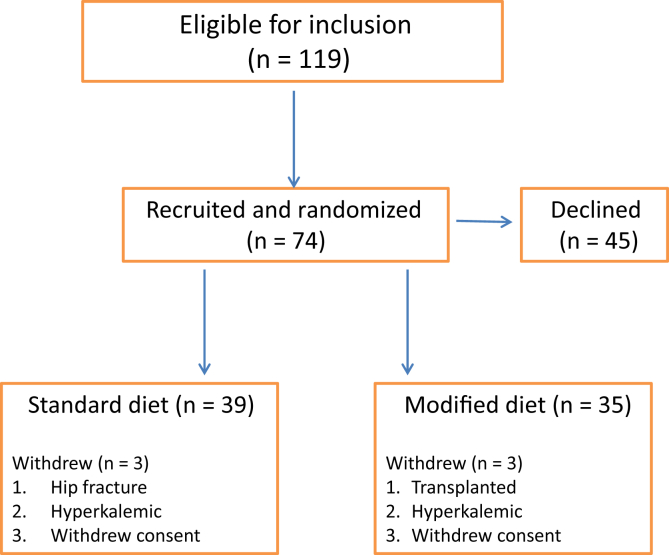
Flow diagram showing recruitment process (inclusion, recruitment, and randomization of study participants).

Figure 1 describes the study flow. Of the 74 participants recruited, 52 were recruited in March 2017, and 22 were recruited in May 2017. In all, 39 participants were randomized to the standard arm and 35 to the modified diet arm. The data presented represent the intention-to-treat analysis of all patients, excluding instances in which an endpoint sample was not recorded. Table 2 summarizes the baseline characteristic of the trial population. The study population was 69% male and predominanately of white ethnicity. There were more patients with diabetes randomized to the modified arm (n = 17) than to the standard arm (n = 10).

**Table 2:** Demographics and medications

Variable	Standard diet (n = 39)			Modified diet (n = 35)		
	n (%)	Median	(Min, max)	n (%)	Median	(Min, max)
Male sex	26 (66.7%)			25 (71.4%)		
White ethnicity	38 (97.4%)			33 (94.3%)		
Diabetes as a comorbidity	10 (25.6%)			17 (48.6%)		
Diabetes as a cause of ESKD	8 (20.5%)			14 (40%)		
Age, yr		59.9	(28.7, 88.3)		61.0	(29.3, 84.9)
Baseline dry weight, kg		81.5	(57.5, 149.1)		80.0	(49, 132)
End of intervention dry weight, kg		80.9	(58, 150)		79.8	(49, 134)
Height, m		1.70	(1.51, 1.86)		1.67	(1.47, 1.87)
BMI, kg/m^2^		28.4	(21.8, 48.9)		27.7	(17.9, 48.4)
IBW, kg		71.2	(57.3, 86.2)		67.7	(49, 83.7)
Recommended protein intake 1.1 g/kg IBW		78.3	(63, 94.8)		74.5	(53.9, 92.1)
Baseline serum phosphate, mg/dl		5.92	(3.53, 12.1)		6.13	(3.90, 10.2)
Baseline urea reduction ratio, %		71.8	(40.4, 84)		70	(58, 83)
Baseline bicarbonate, mEq/l		22	(18, 26)		23.5	(17.6, 29.6)
Medications^a^^,^^b^						
Vitamin D native IU	11 (28%)	800	(343, 7142.9)	10 (29%)	800	(200, 5714)
Alfacalcidol, μg	23 (59%)	0.5	(0.11, 1.0)	20 (57%)	0.37	(0.25, 1.0)
Paricalcitol oral, μg	4 (10%)	2	(1.43, 4)	7 (20%)	1.71	(1, 2)
Paricalcitol i.v., μg	3 (8%)	1.07	(0.86, 2.14)	1 (3%)	2.14	NA
Calcitriol, μg	0 (0%)	NA	NA	1 (3%)	12.5	NA
Cinacalcet, mg	5 (13%)	60	(30, 120)	6 (17%)	60	(30, 120)
Binders^a^^,^^b^						
Sevelamer carbonate, mg	14 (36%)	7200	(800, 9600)	12 (35%)	4800	(1600, 14400)
Sevelamer hydrochloride, mg	9 (23%)	4800	(1600, 7200)	8 (24%)	4800	(2400, 7200)
Calcium carbonate, mg	4 (10%)	1500	(1250, 3000)	1 (3%)	1500	(1500, 1500)
Calcium acetate, mg	12 (31%)	2500	(1000, 4000)	13 (38%)	3000	(1000, 3000)
Calcium acetate magnesium carbonate, mg	1 (3%)	870	NA	0 (0%)	NA	
Lanthanum carbonate, mg	4 (10%)	2625	(1500, 4000)	2 (6%)	3500	(3000, 4000)
Sucroferric oxyhydroxide, mg	3 (8%)	1500	(3, 1500)	5 (15%)	1500	(1500, 1500)
Alucaps, mg	1 (3%)	1425	NA	0 (0%)	NA	

BMI, body mass index; ESKD, end-stage kidney disease; IBW, ideal body weight; NA, not applicable.

a Number of patients on the medication/binder by trial arm.

b Median intake of patients on the medication/binder by trial arm, with associated minimum and maximum amounts.

**Figure 2:**
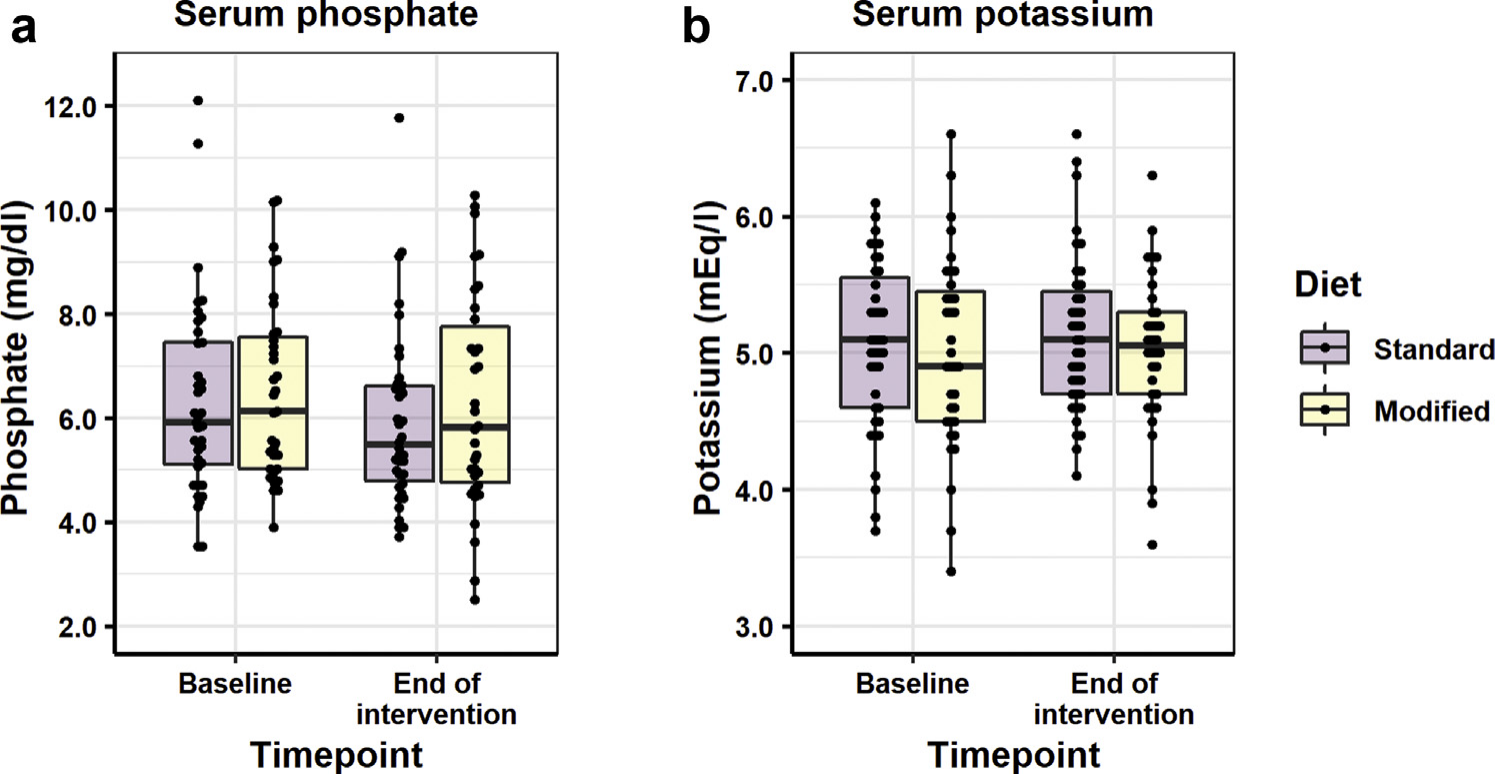
Serum phosphate and potassium. Box plot A shows phosphate at 2 time points, baseline and end of intervention, with the standard diet in the darker shade and the modified diet shown in the lighter shade. The box represents the interquartile range, with the thick line in the box representing median values. In both arms of the trial (standard and modified), there was a small decrease in serum phosphate, likely reflecting education; however, no statistically significant differences were observed for the primary outcome of serum phosphate and potassium between the standard and modified diet. A similar pattern was seen for potassium.

**Figure 3:**
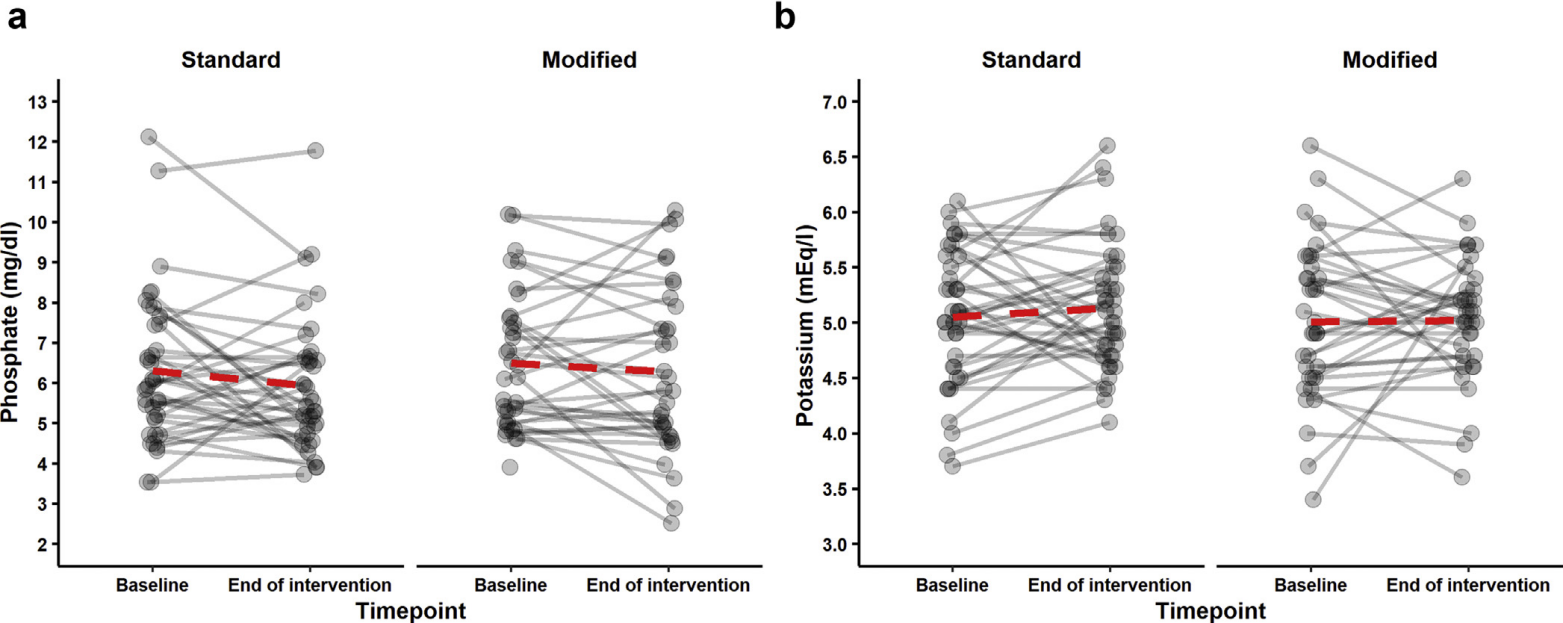
Serum phosphate and potassium. (a) A line is drawn for each subject from baseline phosphate to end-of-intervention phosphate, first for the standard diet and then for the modified diet. (b) A line is drawn for each subject from baseline potassium to end-of-intervention potassium, first for the standard diet and then for the modified diet. At an individual level, there was significant variability in serum phosphate and potassium in both arms of the trial.

Table 3 describes the prespecified trial outcome measures. There was no significant difference in serum phosphate or serum potassium after 1 month between the standard and modified diets (Table 3, Figure 2). Significant variability in serum potassium and phosphate was observed in both groups, as demonstrated in Figure 3. Serum bicarbonate levels were not significantly different in participants following the standard and participants following the modified diet at the end of the intervention (*P* = 0.493) One patient from either arm was withdrawn because of hyperkalemia; withdrawal of the participant assigned to the modified diet occurred at the baseline tests stage, prior to the education step and initiation of the modified diet.

**Table 3:** Main study outcomes

Variable	n^a^		Change from baseline		Analyses to determine impact of diet^b^			
	Standard	Modified	Standard	Modified	Estimate^c^	*P* value	95% CI	
Primary outcome								
Serum phosphate, mg/dl, mean (SD)	38	34	–0.336 (1.536)	–0.295 (1.456)	0.133	0.69	–0.537	0.803
Secondary outcomes								
Total phosphorus intake, mg, mean (SD)	30	30	NA	NA	77.5	0.343	–84.7	239.7
Phytate-bound phosphorus intake, mg, mean (SD)	30	30	NA	NA	207.8	< 0.001	130.4	285.2
Fiber intake, g, mean (SD)	30	30	NA	NA	4.65	< 0.003	1.68	7.61
Intact PTH, pg/ml, median (IQR)^d^	35	31	–3.9 (20.7)	–0.7 (25.0)	0.995^e^	0.968	0.780	1.270
Exploratory endpoint								
C-terminal FGF23, RU/ml, median (IQR)^d^	15	12	–50 (4065)	–105 (2570)	0.98^e^	0.912	0.681	1.141
Safety endpoints								
Serum potassium, mEq/l, mean (SD)	39	34	0.08 (0.60)	0.01 (0.69)	–0.097	0.422	–0.335	0.142

CI, confidence interval; FGF fibroblast growth factor; IQR, interquartile range; NA, not applicable; PTH, parathyroid hormone.

a Number of complete data points available for analysis.

b Serum phosphate and serum potassium were described by multiple linear regression of endpoint explained by covariates of baseline and diet. Intact PTH and FGF23 were described by multiple linear regression of log(endpoint) explained by covariates of log(baseline) and diet. Total phosphorus intake, phytate-bound intake, and fiber intake were described by simple linear regression of intake explained by diet.

c The standard diet was set as the reference level for all linear models.

d The variables intact PTH and FGF23 are displayed nonparametric distributions.

e For log−log transformations, the estimated effect of a change in diet, from standard to modified, is to change intact PTH by a factor of 0.995 and FGF23 by a factor of 0.98, respectively.

On the standard diet arm, there were 3 deviations: 1 change to medication, 1 change to dialysis times, and a new catheter. On the modified arm, there were also 3 deviations: 1 change to medication, 1 change to dialysis times, and 1 change of dialysate.

**Figure 4:**
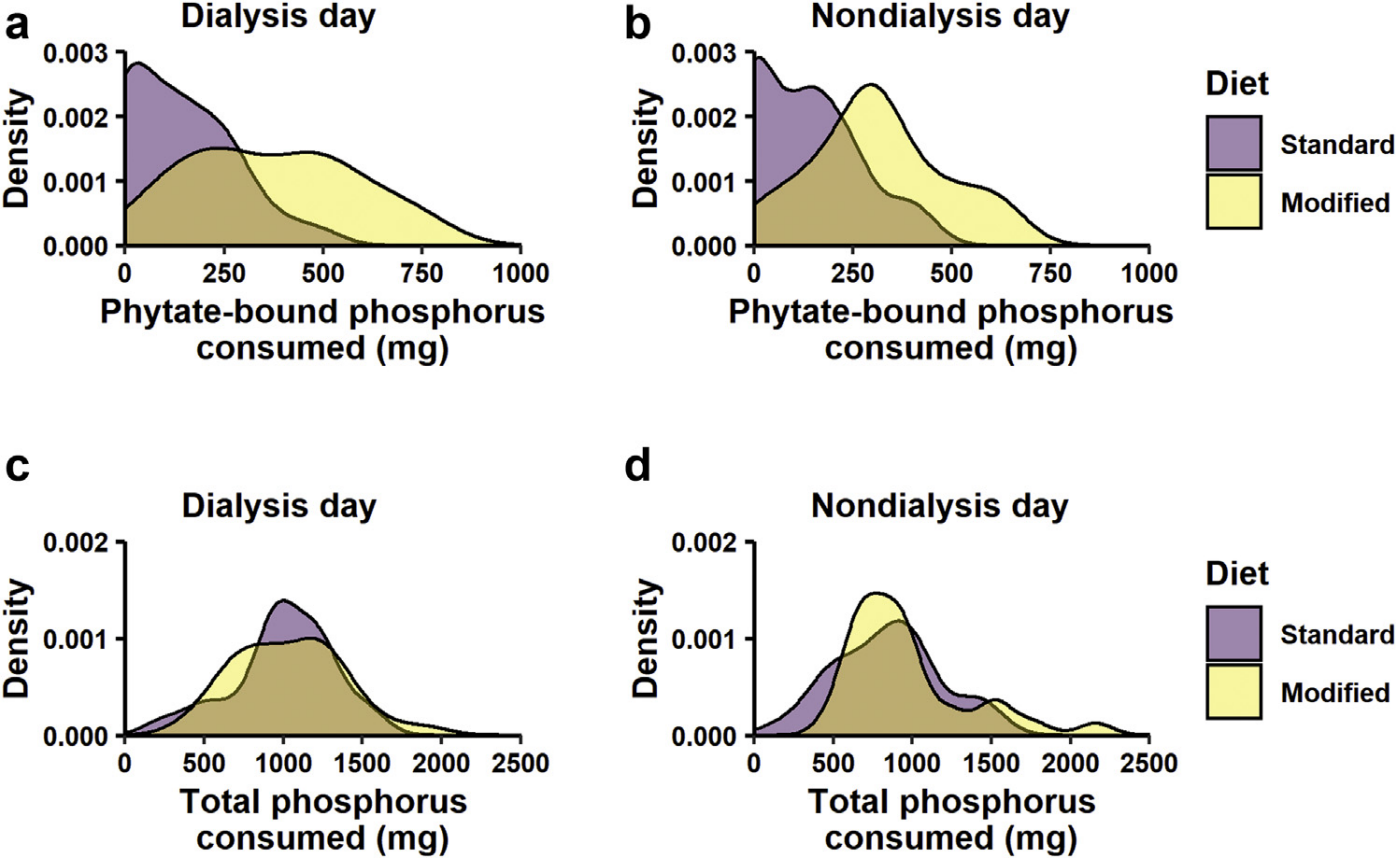
Density estimation of dietary intakes of phytate-bound phosphorus and total phosphorus. The density plot shows the distribution according to food diary entries on (a and c) the dialysis day and (b and d) the nondialysis day. Food diary entries were coded as containing or not containing a significant source of phytate, allowing calculation of both the daily intake of phytate-bound phosphorus and total phosphorus consumed per day.

Table 4 describes mean dietary intake on the dialysis day and the nondialysis day, as assessed by the 2-day weighed intake record at the end of the intervention period. Nutrient intakes were not significantly different between the 2 groups, with the exception of dietary fiber. Although total dietary phosphorus was similar, there were significant differences between the standard and modified diets when we divided the food sources of dietary phosphorus into foods in which phosphorus is or is not bound by phytate (Figure 4). Combining dialysis and nondialysis days, participants following the modified diet consumed more fiber and phytate bound phosphorus than those assigned the standard diet (mean = 4.65 g, 95% confidence interval = 1.68−7.61, *P* < 0.003, and mean = 208 mg, 95% confidence interval = 130−285, *P* < 0.001, respectively).

**Table 4:** Dietary outcome data from end of trial food diaries

Variable	Standard diet (n = 30)		Modified diet (n = 30)		*P* value
	Mean ± SD	(Min, max)	Mean ± SD	(Min, max)	
Dialysis day					
Energy, kcal	1620.6 ± 561.4	(647.4, 3056.2)	1451.8 ± 379	(720.3, 2297.6)	0.18
Fiber, g	14.6 ± 5.9	(3.9, 25.5)	19.2 ± 6.6	(7.1, 34.7)	< 0.01
Sodium, mg	1759.5 ± 747.1	(647.4, 3399.1)	1461 ± 661	(404.5, 3433.7)	0.11
Potassium, mg	1834.9 ± 665.6	(426, 3129.6)	1934.7 ± 700	(797, 3637.8)	0.57
Phosphorus, mg	1012.1 ± 330.6	(207.9, 1584.9)	1038.3 ± 337.9	(391.2, 1887)	0.76
Recommended protein intake, g	78.7 ± 7.57	(63, 94.8)	74.26 ± 10.72	(53.9, 92.1)	0.07
Recorded protein intake, g	71.38 ± 26.44	(14.5, 120.4)	65.79 ± 22.72	(19.3, 118.1)	0.38
ΔProtein, g^a^	–7.31 ± 22.9	(–63.0, –33.7)	–8.48 ± 19.2	(–49.0, –49.3)	0.83
Potential renal acid load, mEq	20.4 ± 13.7	(–18.7, 43.8)	16.7 ± 12.6	(–9.09, 53.2)	0.27
Nondialysis day					
Energy, kcal	1432.4 ± 534.8	(526.5, 2615.3)	1388.7 ± 443.2	(744.9, 2300.6)	0.73
Fiber, g	13.5 ± 6.1	(4.3, 32.4)	18.3 ± 6.1	(5.7, 31.1)	< 0.01
Sodium, mg	1454.8 ± 731.6	(260.3, 4107.2)	1285.5 ± 681.1	(524, 3526)	0.36
Potassium, mg	1813.5 ± 606.3	(686.2, 2800.5)	1923.8 ± 621.4	(1166.4, 3479.8)	0.49
Phosphorus, mg	862.3 ± 328.2	(159.6, 1484.5)	981.8 ± 387.6	(495.4, 2157.6)	0.2
Recommended protein intake, g	78.7 ± 7.57	(63, 94.8)	74.26 ± 10.72	(53.9, 92.1)	0.07
Recorded protein intake, g	62.43 ± 24.27	(16.5, 120)	64.62 ± 21.16	(26.3, 110.8)	0.71
ΔProtein, g^a^	–16.3 ± 22.3	(–61.0, –35.8)	–9.64 ± 21.2	(–61.1, –33.2)	0.24
Potential renal acid load, mEq	13.3 ± 15.1	(–17.4, 44.8)	15.3 ± 11.9	(–9.0, 54.5)	0.57

a Estimates are given in grams (g) of difference between recorded protein intake minus the recommended protein intake.

Reported protein intake was less than the target prescribed in both arms but was not significantly different between arms (Table 4). Mean (SD) reported energy intake (kcals) divided by the basal metabolic rate (BMR) calculated using the Schofield equation was 0.97 (0.32) and 0.86 (0.35) on the dialysis day and nondialysis day, respectively, in participants following the standard diet. It was 0.88 (0.21) and 0.84 (0.25) on the dialysis day and nondialysis day, respectively, in those following the modified diet. There were no significant differences in weight or predialysis urea between the 2 groups. Overall, 26 foods were consumed that contained phosphate additives in participants following the standard diet (14 patients) compared with 22 entries for those following the modified diet (12 patients).

**Figure 5:**
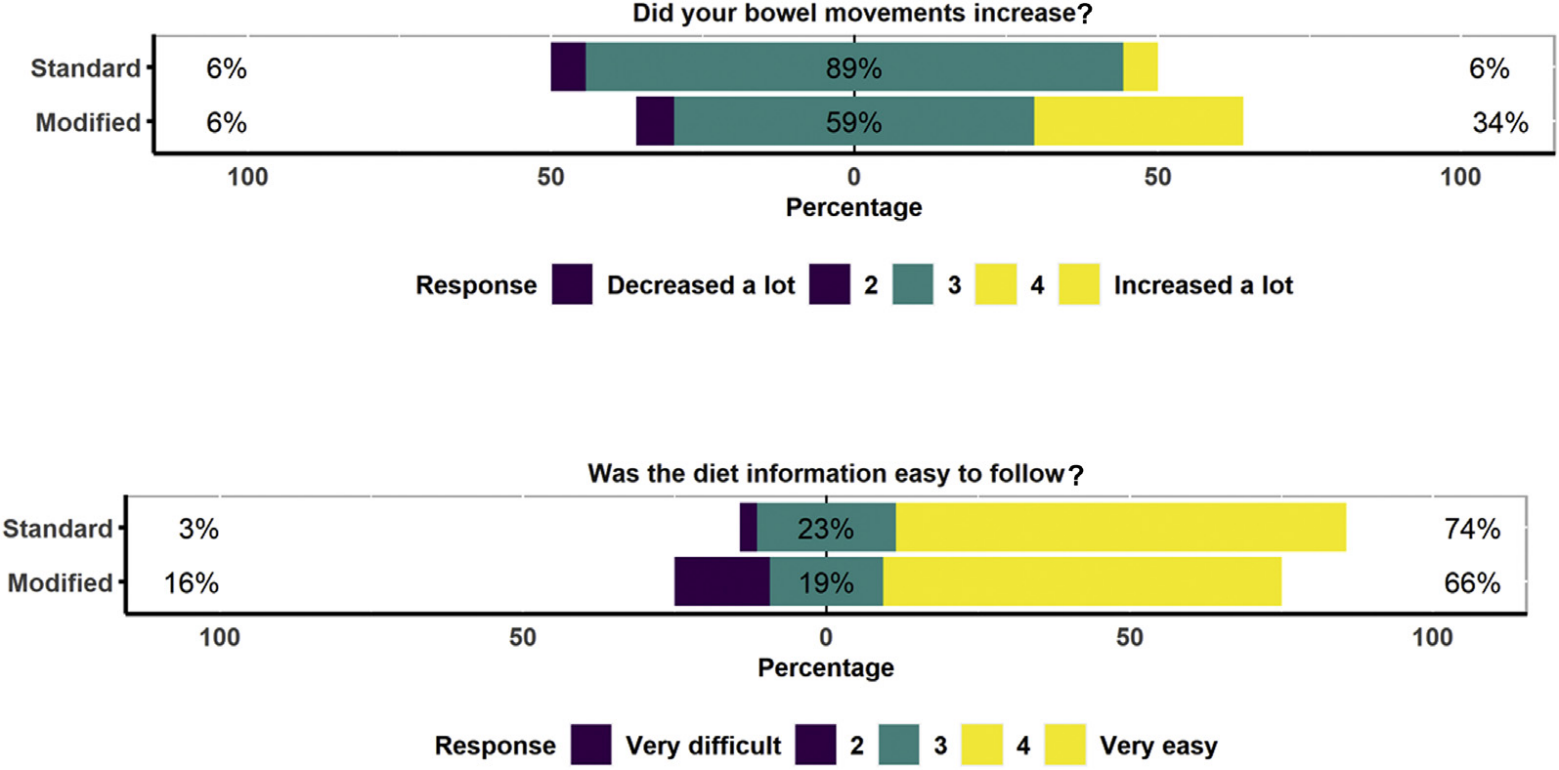
Tolerance data. Participants were asked about their bowel movements and ease of following the information, and were asked to give a rating between 1 and 5.

**Figure 6:**
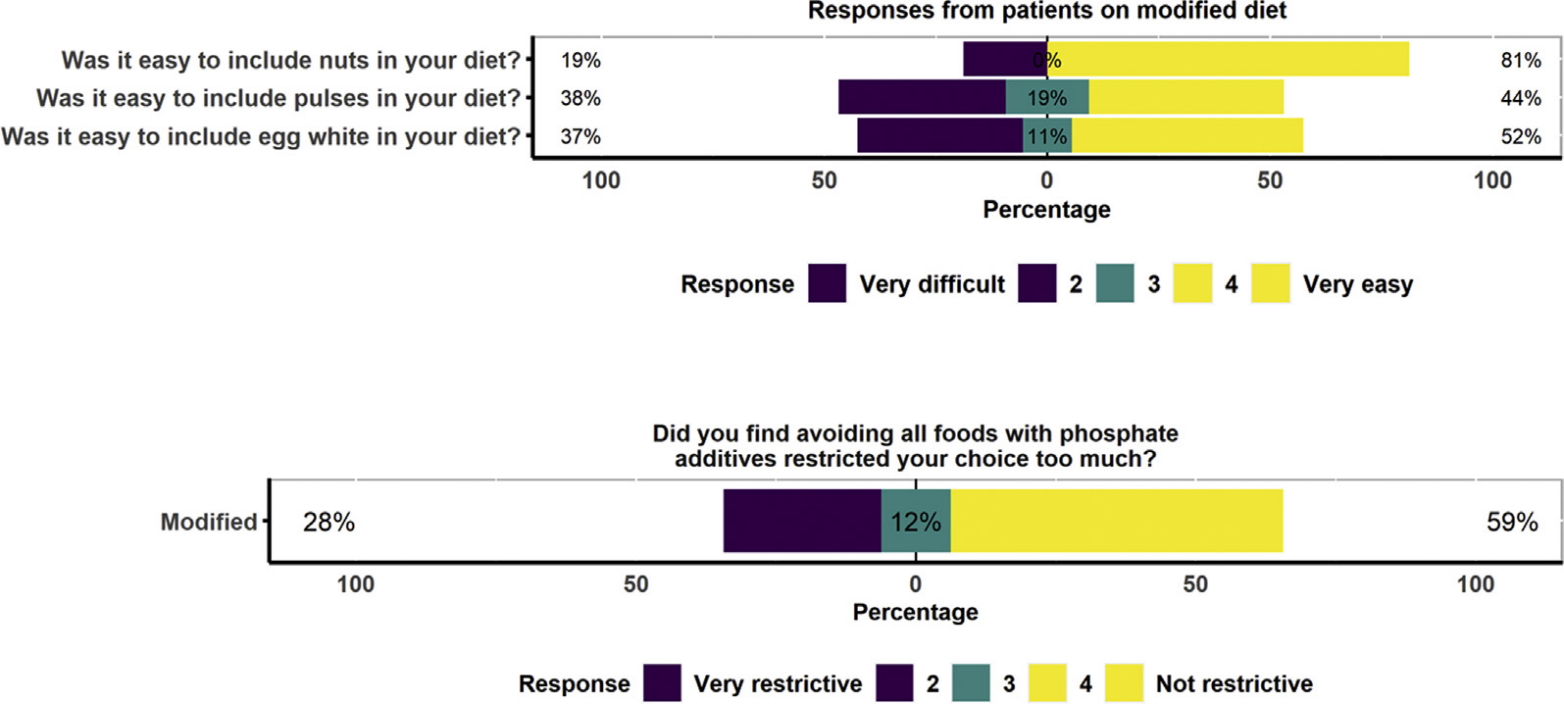
Tolerance data. Participants following the modified diet were ask about the ease of including new foods such as nuts, pulses, and egg whites and about how restrictive it was to avoid all foods with additives, and were asked to give a rating between 1 and 5.

The questions from the tolerance questionnaire are included in Figures 5 and 6. In all, 74% of participants following the standard diet and 66% of those following the new modified diet found the diet easy to follow (*P* = 0.5) (Figure 5). In all, 6% of participants following the standard diet and 34% of those following the modified diet reported an increase in bowel movements (*P* = 0.008) (Figure 5). On the modified diet, 81% of participants found it easy to include nuts, 44% found it easy to include pulses, and 52% found it easy to include egg whites in their diets (Figure 6).

## Discussion

Controlling phosphate load remains the primary goal in the treatment of hyperphosphatemia.^24^ In this study, we tested a modified phosphorus diet. The recommended changes to the national diet sheet summarized in Table 1 were based on a review of the literature and expert consensus. Due to concerns regarding hyperkalemia and dietary potassium intakes, there was caution in the magnitude of change recommended; for example, we recommended a limited amount of pulses and nuts and chose those with the lowest potassium contents, using pulses canned in water.

Neither dietary phosphorus intake nor serum phosphate were significantly different between the modified and standard diets. A possible explanation for the unchanged serum phosphate, is that the dietary change achieved was not of sufficient magnitude to reduce serum phosphate in our study population. We had anticipated that total phosphorus intake would not change because whereas avoiding excessive protein intake, avoiding additives, and reducing phosphorus-to-protein ratios would reduce total phosphorus intake, changing to plant-based proteins and choosing whole grains would increase total phosphorus intake. We were therefore dependent on reduced absorption from phytate-bound phosphorus to effect a change in serum levels. On the dialysis day, patients consumed on average 208 mg more phytate-bound phosphorus while following the modified diet. If we estimate a 30% lower absorption with phytate-bound phosphorus,^25^ then those following the modified diet may have absorbed only 62 mg less phosphorus. Second, the precision of these estimates is limited by the modest sample sizes, especially given the marked variability in serum phosphate levels. There is limited evidence that diabetes may affect phosphate handing, with 1 study in chronic kidney disease (CKD) patients showing that phosphate loading more significantly affected serum phosphate in patients without diabetes.^26^ In our study, we had more diabetics in the modified arm, but the numbers in the trial would not support a subset analysis. Finally the blood samples were taken at various time points during the day and included both fasting and nonfasting samples, which may influence serum phosphate levels.^27^^,^^28^ Future trials should consider a larger sample size with longer follow-up and more uniform timing of samples.

Intakes of phytate-bound phosphorus were significantly higher in participants following the modified diet. This is important, because it indicates that patients did change their diets and that there was good separation between the 2 trial arms. Although the mean dietary potassium intake was higher in participants following the modified diet, the difference was not significant, and comparable serum potassium control was maintained across the 2 groups. The risk of hyperkalemia from increasing dietary potassium intake from whole grains and plant-based protein in ESKD needs further investigation.

Protein intakes were lower than recommended in both arms of the trial, but may be explained in part by underreporting. Such underreporting is common in dietary surveys,^29^, ^30^, ^31^ but is unlikely to be of a differential magnitude between our study arms, thus allowing us to compare the 2 diets. There are also concerns in the literature regarding the accuracy of phosphorus content in standard food tables, particularly in foods containing phosphate additives.^32^^,^^33^ As the level of consumption of foods containing phosphate additives in this study was low overall, this may not be a significant limitation.

Fiber intakes were significantly increased in the modified diet, although they were still not sufficient to meet general population recommendations. The increase in fiber intake is supported by the observation that patients’ bowel movements increased in those following the modified diet. This is clinically significant, because constipation has been reported in up to 72% of dialysis patients.^34^ An increase in fiber intake has also been shown to reduce levels of uremic toxins in CKD and ESKD.^35^^,^^36^ Uremic toxins may contribute to poorer outcomes.^37^, ^38^, ^39^, ^40^ Although various interventions such as increasing fiber may alter the composition of intestinal microbiota in CKD and ESKD and reduce uremic toxins, clinical benefit is not yet confirmed.^39^^,^^41^

In this study, patients were encouraged to exchange 2 animal-based protein exchanges such as a 50-g meat portion for 2 plant-based protein exchanges such as 200g of pulses. The year 2016 was declared the International Year of the Pulse to heighten public awareness of the nutritional benefits of pulses,^42^ and these benefits may extend to the CKD population.^43^ Results from different studies seem to confirm a kidney-protective effect of plant-based diets,^44^, ^45^, ^46^ and recent reviews suggest that liberalizing the diet to allow plant foods may significantly improve the health and well-being of CKD and ESKD patients.^8^^,^^47^ There is also interest in the potential benefit of phytate in the attenuation of cardiovascular risk in CKD populations,^48^ with a recent study demonstrating that an i.v. formulation of phytate significantly attenuated the progression of coronary artery calcium and aortic valve calcification in patients with ESKD receiving hemodialysis.^49^

It has been reported that 55% of dialysis patients recalled “feeling deprived with dietary restrictions.”^50^ This modified diet introduced pulses, peas, and nuts as well as relaxed restrictions on whole grains. Increasing choice was a strong reason to guide adoption.

The strengths of our study are as follows. First, we tested a more liberalized plant-based diet in a multicenter RCT. Second, the intervention was tested in a hemodialysis population in whom hyperkalemia is more of a concern. Third, the study included dietary assessment using weighed diet records, providing valuable nutrient level information. Finally, this pragmatic trial was undertaken by registered dietitians who, with support from nephrologists, demonstrated the potential to carry out high-quality research at the front line. Limitations of this pilot study include its short duration and the modest sample size. Although 1 month allowed us to assess the short-term efficacy, safety, and tolerability, it did not allow us to assess the long-term effect and sustainability.

As renal health care professionals, our discussions have focused on nutrient-based recommendations: for example, high protein, low phosphorus, low potassium, and low salt. The role of the renal dietitian has always been to translate multiple nutrient restrictions into individualized, practical, food-based recommendations. The dietitian aims to achieve this within a balanced healthy diet while maintaining quality of life. This is a complicated and evolving science and one that requires significant research.^51^^,^^52^ In this trial, we tested food-based recommendations, individualized to meet each patient’s requirements. Although the focus was only on phosphorus, all other nutrient restrictions were considered and incorporated into the prescribed diet.

Recently, nutrition science has been moving toward dietary patterns or food-based recommendations, with dietary patterns such as the Mediterranean diet and the new Nordic diet gaining credence in people with kidney disease.^53^^,^^54^ Dietary patterns may be more predictive of disease risk than individual foods or nutrients.^55^ We eat food, not nutrients, and, as demonstrated by dietary phosphorus that is phytate bound, the matrix in which food is digested has a huge bearing on absorption. Educating patients in using dietary patterns such as the Mediterranean diet pyramid^54^^,^^56^ may be an adequate dietary management strategy in the earlier stages of CKD; however, in the later stages of CKD and ESKD, the prescribed dietary pattern will need to be more carefully individualized because of concerns regarding intakes of specific nutrients (in particular, potassium) and because of the need to individualize protein requirement based on the patient’s stage of disease and treatment prescribed. The suggested changes, tested at trial, move our recommended renal diet closer to the Mediterranean dietary pattern while retaining the ability to individualize nutrient restrictions.

Clinical Practice Guidelines for Nutrition in CKD, which are being jointly undertaken by National Kidney Foundation’s Kidney Disease Outcomes Quality Initiative (KDOQI) in collaboration with the US Academy of Nutrition and Dietetics, are due for publication in 2020. These nutrient-based guidelines should be translated into food-based recommendations like our individualized diet plan and tested in an RCT trial. Undertaking these trials is undoubtedly challenging,^57^ but is absolutely essential to demonstrate the effectiveness of dietary interventions and to improve dietary strategies in CKD and ESKD.^58^ Research in dialysis patients is particularly important because of concerns regarding hyperkalemia. Although this advice may need to be used more cautiously in hyperkalemic patients, our study in a dialysis population provides an important first step toward safely reducing restrictions and exploring the effect of a specific dietary pattern on biochemical parameters and tolerance. The current study shows the feasibility of conducting a large-scale, longer-duration trial to demonstrate the efficacy of dietary intervention.

## Disclosure

All the authors declared no competing interests.
